# Environmental regulation and high-quality agricultural development

**DOI:** 10.1371/journal.pone.0285687

**Published:** 2023-05-12

**Authors:** Yutong Leng, Xinmin Liu, Xinjiang Wang

**Affiliations:** 1 School of Economics and Management (College of Cooperatives), Qingdao Agricultural University, Qingdao, China; 2 Qingdao Agricultural University, Qingdao, China; Southeast University, BANGLADESH

## Abstract

The key trend for future agricultural growth is efficient, green and sustainable high-quality development, and it is crucial to sort out the factors influencing high-quality agricultural development.

**Objectives**: The purpose of this study is to dissect whether environmental regulation has a catalytic effect on quality rural development, through which paths it is driven and whether there is a threshold effect to be further clarified.

**Method**: In this study, the panel data of 34 provinces in China from 2012 to 2018 are used, and 17 variables are used to construct an evaluation index system, covering four dimensions: agricultural endowment, agricultural output level, agricultural green degree and social sustainability. The high-quality development of agriculture is measured by entropy method. And further, using a baseline regression model and a mediating effects model, we empirically investigated the impact of environmental regulation on high-quality agricultural development and its mechanism of action, and empirically assessed the nonlinear effects of environmental regulation using a threshold regression model.

**Results**: Environmental control, as noted in the study, considerably assists in the establishment of high-quality agriculture; at the same time, large-scale land management plays a role in mitigating the influence of both. There is a single income threshold impact on rural households between high-quality agricultural growth and environmental regulation, and once that threshold is reached, the influence of high-quality agricultural growth grows.

**Contribution**: According to the research findings, recommendations are made for the design of scientific environmental regulation policies, the establishment of a sound service system for large-scale rural land management, and the establishment of a stable mechanism for rural residents to sustain their income, in order to strengthen the effect of environmental regulation and realize high-quality agricultural growth in China. The marginal contribution of this paper is to enrich the study of the relationship between environmental regulation and high-quality agricultural development, which has theoretical and practical implications for promoting sustainable agricultural development.

## Introduction

Agriculture is the foundation of human clothing, shelter, food, survival, transportation, living water, and development. Whether agriculture can maintain quality and incremental development is related to economic development, social stability, and national rejuvenation, and its importance is self-evident [1]. The traditional agricultural production model of backward resource development technology, large doses of chemical fertilizers, and high concentrations of pesticides focus on the role of agriculture only on economic contribution [2]. The coexistence of low efficiency and high pollution ultimately leads to multiple serious problems such as excessive greenhouse gas emissions, loss of biodiversity, food pollution, degradation of soil quality, and gradual fragility of the ecological carrying capacity. As a result, it is not sustainable [3–6], and the conflict between development and conservation is becoming increasingly prominent. Between reform and opening up and the beginning of the 21st century, China’s crude development approach sowed the seeds of economic inefficiency, resource shortages and environmental damage. Economic transformation is a process requiring continuous productivity growth with significant technological advancement drive [7]. Now China is the second-largest economy in the world [8], and while it has achieved world-renowned achievements, problems such as resource shortages, saturated environmental carrying capacity and unsustainability have come to light [9].

With the changes in resources, environment and socio-economic situation, the agricultural development pattern of each country has also taken on new dynamics, with agricultural development not only focusing on "quantity" but also adapting to the needs of society and people, thus achieving a "quality" transformation [10]. Sustainable development is a process that requires simultaneous global progress in economic, human, and environmental dimensions as well as technological advances. The 2018 Nobel Prize in Economic Sciences discusses studies such as green development for long-term economic growth through sustainable long-term economic growth in studies conducted in the 1970s [11]. The ultimate goals of productivity improvement are greater competitiveness, higher profitability, higher living standards, and better economic and social prosperity [12]. Agriculture, as a key component of high-quality growth [13], effectively reflects the country’s strategic thinking and transformation of agricultural development, signifying a greater pursuit of quality and efficiency [14]. In addition, high-quality agricultural development also plays an irreplaceable strategic role in promoting the integration process of rural and urban areas, safeguarding the rights and interests of the majority of farmers and achieving common prosperity. Therefore, promoting agriculture (efficient, green and sustainable growth) is essential for China to enter a new era, as well as a key to ensuring national food security and other major issues.

A crucial practical question is how Chinese agriculture can improve the efficiency of resource use, accomplish green growth, reduce pollutant emissions, low-carbon development, and circular development [15] and ultimately achieve high-quality development. However, it should also be noted that breaking the existing production model to find a new way out requires farmers, agricultural enterprises and other agricultural actors to pay a cost, and it is far from enough to rely on the power of the market alone. Governments and international organizations must introduce new science-based regulatory frameworks [16]. In China, the sustainable development strategy dates back to 1992, with the introduction of the Agricultural Law 1993 to prevent agricultural pollution. From year of 2000 to 2010, several regulations and laws were enacted or amended, including the Soil and Water Conservation Law, the Air Pollution Prevention and Control Law, and the Cleaner Production Promotion Law. The relevant laws and legislations have also made detailed and clear provisions on the use of pesticides in low doses and concentrations, optimizing the use of arable land resources and conserving water resources. Agricultural environmental regulations play a crucial part in fostering the high-quality growth of Chinese agriculture. What is the relationship between the two? What are the transmission mechanisms through which they work?

Much of the research that has been done examines the role of environmental regulation on agriculture from two perspectives. On the one hand, scholars have found that environmental regulation has a significant positive impact on green technology innovation and that innovation under environmental regulations is a driver of total factor productivity in agriculture [17]. On the other hand, environmental regulations are also considered as an important factor in promoting green development in agriculture [18]. Reasonable environmental regulation can promote the upgrading of local agricultural industry structure and technological innovation, and reduce agricultural pollution and agricultural carbon emissions [19]. However, strict environmental regulation will be higher than the input costs of agricultural development, which will not only fail to improve agricultural inefficiencies, but will instead exacerbate agricultural pollution in both local and neighboring areas [20, 21], thus ultimately inhibiting the greening of agriculture in local and neighboring regions. Currently, China fully realizes the development of comprehensive green transformation of agriculture [22] and the Chinese economy has made progress from the stage of high speed growth to the stage of high-quality development [23, 24]. In summary, existing studies have focused more on the use of environmental regulations for total factor productivity in agriculture and green development in agriculture, but few studies have explored the use of environmental regulations for high-quality agricultural development. To explore the intrinsic link amongst agricultural quality development and environmental regulation, this paper compares the existing research results and develops an evaluation system of China’s agricultural quality development indicators. The innovative points of this paper are: (1) To construct an evaluation index system for high-quality growth of agriculture in China from 17 indicators in four dimensions: resource endowment, efficiency, greenness, and sustainability, and to measure and evaluate the level of high-quality growth of agriculture in 34 Chinese provinces from 2012 to 2018; (2) Based on the perspective of land scale, we analyze the possible paths through which environmental regulation has an impact on the development of agriculture, clarify the mechanism between them, and provide empirical evidence for the theoretical view that environmental regulation promotes land scale and thus China’s high-quality growth of agriculture; (3) Utilizing a threshold regression model, we investigate the non-linear correlation amongst environmental regulation and high-quality agricultural development. Through a threshold regression model, we analyze the influence of environmental regulation on the growth of agriculture at different levels of farmers’ income, for the purpose of offering a foundation for local governments to promote efficient, green and sustainable development in China according to different income levels; (4) Total factor productivity is used as an indicator of environmental regulation, and an integrated approach based on parametric analysis is used for parametric analysis to fill the model gap between research on total factor productivity and high-quality agricultural development.

## Literature review and hypothesis

Before the topic of high-quality agricultural development was raised, many scholars focused their research perspectives on agricultural production efficiency. In early academic studies of agricultural production efficiency, only the effects of capital and labor inputs on output were generally considered, ignoring the effects of pollution emissions on agricultural ecology and production efficiency in the actual production process. Uthes S. et al [25] constructed indicators based on competitiveness, rural viability, environment, and equality of expenditures to assess the implementation of rural development measures and found that the more competitive experimental group was able to increase agricultural productivity higher but the trend of data on environment was not satisfactory. The influence of ecology as an increasingly important factor on agricultural efficiency is becoming more and more important, and thus many scholars have analyzed the factors influencing agroecological efficiency. In terms of human capital, Hou, Mengyang et al. pointed out that rural labor migration has a significant contribution to improving the eco-efficiency of food production [26]. Li Lu et al. pointed out that the aging of the rural population has a negative impact on agroecological benefits [27]. In addition, the low skills of agricultural laborers and inefficient land laborers and inefficient land use are also responsible for efficiency losses [28].

As a result of the administration’s determination to protect the natural world, formal environmental legislation refers to rules or standards pertaining to environmental preservation that are created with government agencies as the dominant authority and subject to public power. It specifically covers environmental assessments, ecological monitoring, contamination enforcement, and emission limits for wastewater and waste gas [29]. Growth in the economy and environmental quality have an inverse U-shaped relationship. Thus, pollution rises during the early phases of economic expansion and then starts to fall after the growth rate exceeds a specific point. Thus, according to the EKC hypothesis, there may also be an inverted-U relationship between the impact of environmental rules and green total-factor productivity. Environmental regulations primarily affect GTFP through two main mechanisms: the "regulatory cost" effect and the "innovation reimbursement" effect. The Porter theory, which contends that rational environmental standards policies will boost the opportunity of businesses to create something new, strengthen their technology and innovation, and then boost their efficiency, compensate the rising costs, and boost their financial performance, is the foundation of the "advancement remuneration" effect. Based on neoclassical economic theory, the "cost-of-compliance" impact postulates that environmental legislation will raise the cost of environmental regulation, displacing enterprises’ expenditure in research and development and innovation, leading to poorer efficiency and smaller profit margins. In particular, rigorous environmental regulation will boost the pressure and incentive for businesses to develop and attain technical advancement during the initial stages of formal environmental legislation. Yet, the expansion of the green economy would necessitate a sizable amount of inventive development due to the gradual tightening of environmental rules [30], which will have scale implications. Enterprises may divert funds from development and innovation performance to reduce costs and preserve their current profit levels as the expense of environmental regulation and the resource allocation will increase. The "conformity cost" effect currently outpaces the "innovation reimbursement" effect. Enterprises will examine easy ways to cut emissions in order to comply with environmental regulations, which will discourage them from innovating and prevent the growth of green total-factor productivity levels.

**Fig 1:**
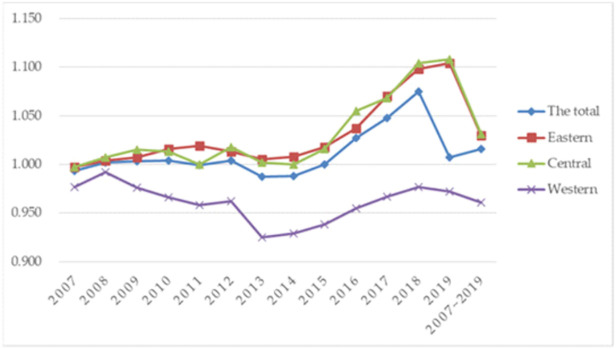
Green total-factor productivity (GTFP) in different regions of China.

In this study, we compare the changes in GTFP in the regions around cities by dividing them into eastern, central, and western regions. Fig 1 shows the comparative differences of GTFP between different regions. The range of GTFP swings between 0.9 and 1.15, with a very moderate change, but there is a regional-crossover difference across various years. Up till 2019, the eastern and central regions are both above the total level while the western region is below the total-level line. The center region is greater than the eastern region. While the shift in GTFP in the western region has a lag and is a little bit slower than the development in GTFP in the eastern and central regions, they are both broadly in line with the overall level. This is likely due to the eastern region’s advantageous geographic location, high degree of technological as well as scientific innovation, and concentration of high-tech enterprises, which are better suited to support the development of the green industrial structure and increase GTFP. Because it is located inland and lacks the knowledge and technology to create a green economy, the western region has largely concentrated on conventional heavy industry and industries with excessive intake, high contamination, and high emissions. As a result, the advancement of GTFP in the western province has lagged behind that of the eastern and central regions.

As changes in consumer demand and thus the structure of agricultural production [31] are driven, the resource-factor-driven economic growth model is gradually being replaced by the mainstream model of high-quality economic progress [32], making the promotion of high-quality growth of agriculture an essential theme for future progress. However, breaking away from the existing agricultural production model and seeking high-quality growth of agriculture requires a great deal of effort, and it is difficult to achieve this by market means alone, so the power of the government is particularly important. In the case of agricultural production, government power is reflected in the legal policies’ development related to the protection of the environment. Environmental regulation thus means, specifically, government regulations that guide and constrain farmers in order to urge them to participate in the mitigation of rural environmental pollution and to use green technologies for production on their own initiative [33].

In the actual production process, farmers’ behavioral decisions are indeed influenced by environmental regulation [34]. According to the conventional idea, which is based on neoclassical economic theory, when farmers’ behavior is first regulated by various laws, producers are often faced with the problem of eliminating the consequences of pollution and increasing research and development of new and emerging technologies. They often need to invest more money for resolving this issue. It is because of this increased capital investment that less and less investment is made in other profitable projects, creating a "crowding out" effect. Assuming that other external conditions do not change, the pressure of environmental regulation can only increase the "cost of loss" for agricultural operators, crowding out development funds and thus negatively affecting agricultural development’s quality, that is, the "compliance cost" effect of environmental regulation [35]. However, environmental regulation can also lead to induced technological change and promote technological progress [36], and moderate environmental regulation policies can stimulate agricultural producers to take the initiative to improve the quality of their products, reduce costs and seek innovation, thereby generating "compensation for gains" [37]. In the long run, as they are encouraged by their income, they will spontaneously use green technologies in their agricultural activities, optimizing the allocation of factors at an efficient level. This gradually spreads from a partial to a "cost of compliance" overall, offsetting in turn the "innovation compensation" impact of environmental regulation [38].

Therefore, the policy will initially have a "crowding out" effect, but after a certain period of time, the high demand for clean technology and the market pressure for survival will force improvements in agricultural production methods. The reduction of high-pollutant inputs and low agricultural output will ultimately lead to increased competitiveness. The capital gains from this forced innovation of agricultural agents can far offset the initial input costs [39], ultimately making economic growth and environmental protection complementary and realizing the high-quality growth of Chinese agriculture. Hence, in this paper, hypothesis 1 is put forth.

H1: Environmental regulation can effectively contribute to high-quality agricultural development.

China’s agriculture is based on smallholder farmers, with National Bureau of Statistics data indicating that as of 2019 over 70% of arable land is still cultivated by scattered smallholders. As they are tempted by the huge demand for food, smallholder producers have been using excessive amounts of chemical fertilizers to obtain high yields, and such production undermines the nation’s environmental sustainability and food production security [40]. Moreover, urban area expansion and rapid urbanization have also resulted in the depletion of arable land resources [41], which is even more detrimental to large-scale and sustainable agricultural operations. The land is widely regarded as the most basic means of production for agricultural development, but the fragmentation and decentralization of land in China are considered to be a major obstacle to agricultural development, and its overall condition of "large population and small land" [42] dictates the need to achieve moderate scale land management as soon as possible to improve agri-cultural productivity in order to avoid food problems [43].

In order to improve the current situation, the state has implemented several policies related to environmental regulation for alleviating agricultural pollution and improving farmland’s quality [44], such as the Regulations on the Implementation of the Land Management Law of the People’s Republic of China as well as the Land Management Law of the People’s Republic of China and so on. "Conserving and concentrating land and strictly guarding the red line of arable land" is an important decision to protect arable land. The decision is the cornerstone of steady societal and economic development. With the implementation of the policy and the acceleration of industrial restructuring, the scale of land transfer has increased annually. Contrary to the previous fragmented and rough management methods, environmental regulations have provided favorable conditions for the organization, specialization, standardization and socialization of agricultural production, and mechanized farming has been gradually realized in some places through the transfer of land [45]. From the perspective of scale economy theory, moderate scale operation of land can improve agricultural productivity and fertilizer utilization rate, making the input factor ratios more scientific, especially the reduction of polluting factors such as fertilizer and other polluting factors to increase efficiency [46], and also reduce the negative impact of land abandonment and idleness on agricultural surface pollution, promoting agriculture towards the aim of high-quality growth of overall increase in quantity, quality and efficiency. Therefore, hypothesis 2 is proposed in this paper.

H2: Land-scale management mediates between environmental regulation and high-quality agricultural development.

Farmers dominate the development of Chinese agriculture, and their behavior determines whether agriculture can move towards improved quality and increased quantity. For farmers, the rise in the intensity and the number of various policies have led to a constant need for farmers to pay money to accomplish their goals, such as the cost of purchasing clean production factors, cost of recycling waste, the cost of purchasing or upgrading agricultural machinery and equipment, and the cost of environmental restoration [35]. The rising cost of environmental remediation not only crowds out other agricultural producers’ cost expenditures, particularly the investment in R and D of green production technologies, which has the problems of large initial capital investment, slow results and long waiting period, but also increases the burden of agricultural producers [47]. The rise in the intensity of various policies will cause agricultural producers to decrease the intensity of utilization of input factors such as pesticides, which are inefficient and have high pollution emissions, and this will have an impact on the profitability of agricultural producers [36]. Regarding the "compliance cost" impact, environmental regulations raise capital investment in the agricultural production process and reduce the profitability of agriculture. Therefore, the level of farmers’ income is crucial to the effectiveness of environmental regulations, and when farmers’ income levels are low, there is greater resistance to environmental regulations due to concerns about the costs of new, green and sustainable agricultural production methods. When farmers’ income levels are higher, farmers will be more confident and motivated to change their production methods, and there will be less resistance to the promotion of environmental regulations, which will have a stronger impact on the development of high-quality agriculture. Therefore, in this paper, hypothesis 3 is proposed.

H3: The impact of environmental regulation on quality agricultural development has a threshold effect.

**Fig 2:**
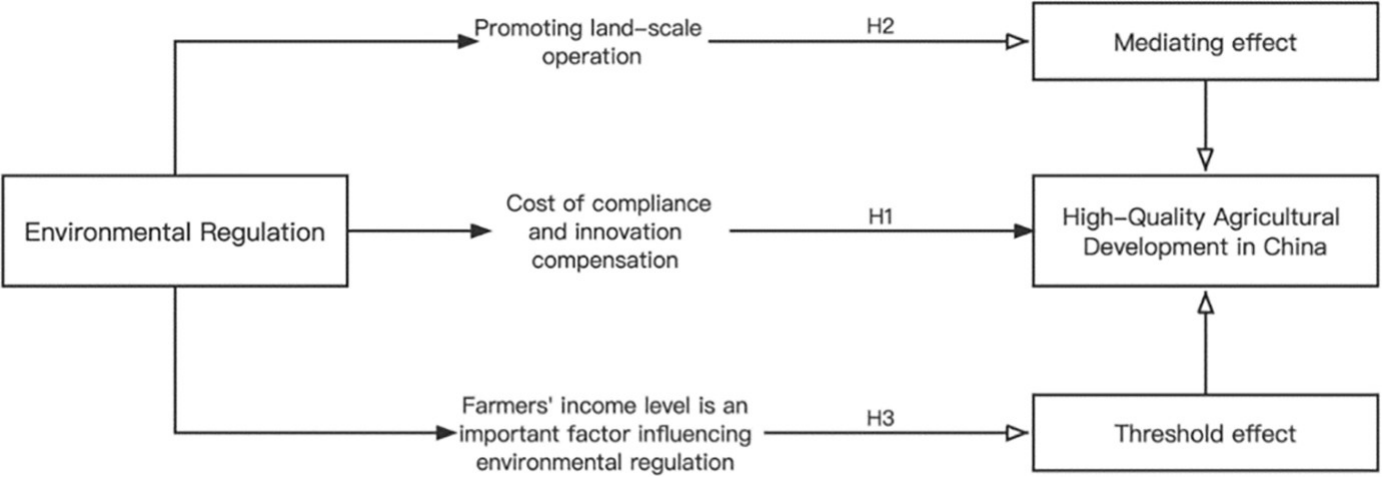
Research design framework.

The research design framework is shown in Fig 2.

## Study design

### Variable selection

#### Interpreted variable

Quality Development in Agriculture (Quality). Scholars have used the measure of TFP or total factor productivity to gauge the quality of agricultural development. However, total factor productivity, due to its single indicator, is not equivalent to the indicators of quality development [48] and is not sufficient to complete a comprehensive summary and generalization [49]. In this paper, we summarize previous research that "high-quality" is an innovation that aims to satisfy people’s aspirations for a better and upgraded life according to the current economic development, with more emphasis on multidimensional coordination as well as economic, ecological, environmental and social. In order to estimate the quality of agricultural growth using four basic indicators and 17 supplementary indicators, this study develops a thorough index system for high-quality growth of agriculture namely, agricultural endowment, agricultural production level, agricultural greenness, and sustainable social development, and utilizes the entropy value approach for computing China’s agricultural quality index through entropy value and weighting. Index and the measurement method of each indicator is shown in Table 1.

**Table 1:** Evaluation index system for high-quality agricultural development.

Level 1 Indicators	Level 2 Indicators	Level 3 Indicators	Measurement Method	Direction
High-quality agricultural development	Resource Endowment	Seeded area per capita	Total sown area of crops / year-end population of the region’s villages (ha/person)	+
		Water resources per capita	Water resources per capita (m3/person)	+
		Forest resources	Forest cover /%	+
	Efficiency	Labor productivity	Total output value of agriculture, forestry, animal husbandry and fishery / rural employed population (ten thousand yuan/person)	+
		Land output rate	Total agricultural output value/crop cultivation area (100 million yuan/thousand hectares)	+
		Arable land utilization rate	Sown area/cultivated area /%	+
		Effective irrigation rate	Effective irrigated area/sown area/%	+
		Degree of agricultural mechanization	Total machinery power / arable land area (kW/ha)	+
	Green	Diesel Utilization	Value added of primary industry/diesel use (100 million yuan/10,000 tons)	+
		Water utilization rate	Value added of primary industry/water use in agricultural production (100 million yuan/100 million cubic meters)	+
		Fertilizer use per unit area	Fertilizer application/sown area (tones/thousand hectares)	-
		Amount of agricultural film pollution per unit area	Agricultural film pollution / sown area (tons/thousand hectares)	-
		Amount of pesticide use per unit area	Pesticide Usage/Sown Area (tones/thousand hectares)	-
	Sustainability	Urban-rural differences	Disposable income of urban residents / disposable income of rural residents / %	-
		Consumption level	Consumption level of rural residents (ten thousand yuan / person)	+
		Degree of environmental management	Proportion of nature reserves in the area under its jurisdiction /%	+
		Farmers’ fixed investment	Total fixed investment of rural households / Rural population (100 million yuan)	+

The entropy value method is calculated in the following steps.

The unit of measurement as well as the direction of each indicator is not uniform, and the data needs to be standardized to remove the impact of the dimension. Among them, the positive indicators reflect the positive effect and the negative indicators reflect the negative effect. The standardization model is as follows.

Step1: (1) This formula is used to adjust the positive indicators.
$$Y_{ij}=\frac{X_{ij}-MinX_{ij}}{Max\left(X_{ij}\right)-MinX_{ij}}$$ (1)
  (2) This formula is used to adjust for negative indicators.
$$Y_{ij}=\frac{\text{Max}\left(X_{ij}\right)-X_{ij}}{\text{Max}\left(X_{ij}\right)-MinX_{ij}}$$ (2)
  In Eqs 1 and 2, among them, *Y*_*ij*_ is the standardized value of the j year of the I index (i = 1,2,3,…,30; j = 1,2,3,…,17), *X*_*ij*_ represents the original data of the index value of the j year of the I indicator, *Min*(*X*_*ij*_) and Max(*X*_*ij*_) represent the minimum and maximum values of item j.
Step2: This formula is used to calculate the sample weights.
$$P_{ij}=\frac{X_{ij}}{\sum_{i=1}^{n}X_{ij}}$$ (3)
  *P*_*ij*_ represents the share of the ith country in the indicator under the jth indicator, (i = 1,2…,30; j = 1,2…,17).
Step3: This formula is used to calculate the entropy values.
$$e_{j}=-K\sum_{i=1}^{n}P_{ij}\;\text{ln}(P_{ij})\;(k>0,\;\;k=\frac{1}{\text{ln}\left(n\right)},e_{j}\geq 0)$$ (4)
  *e*_*ij*_ represents the entropy value of the jth indicator.
Step4: This formula is used to define the coefficient of variation.
$$g_{j}=\frac{1-e_{j}}{m-E_{e}}\;(E_{e}=\sum_{j=1}^{m}e_{j},0\leq g_{i}=1,\sum_{j=1}^{m}g_{j}=1$$ (5)
  Calculate the coefficient of variation of the jth indicator. For the jth indicator, the greater the difference in indicator values, the lower the entropy value.
Step5: This formula is used to calculate entropy weights.
$$w_{j}=\frac{g_{j}}{\sum_{j=1}^{m}g_{j}}\;\left(1\leq j\leq m\right)$$ (6)
  *w*_*j*_ is the calculated weight.
Step6: This formula is used to calculate the agricultural quality development index.
$$Quality_{i}=\sum_{j=1}^{m}w_{j}Y_{ij}$$ (7)
  *Quality*_*i*_ represents a comprehensive score, which is to calculate the final index of high-quality agricultural development.

#### Explanatory variable

Environmental Regulation (ER). No unified standard is available for measuring this concept. With the introduction of global warming, carbon emissions, an important cause of global warming, have been adopted by many scholars as an important measure of environmental regulation in agriculture. This paper draws on this idea and chooses to measure carbon emissions per unit of sown area. In this paper, the carbon emissions from agricultural inputs such as fertilizer and mulch, the carbon emissions from energy consumption during agricultural production such as diesel, and the carbon loss from soil tillage and irrigation are taken into account in previous studies. In this paper, the IPCC carbon emission factor method is used, which is based on the above six types of carbon inputs multiplied by their carbon emission factors. Its calculation formula is:

$$C=\sum C_{i}=\sum N_{i}\times K_{i}$$ (8)

where the weights are given by the average value shares as follows:

*C* denotes the total agricultural carbon emission

*C*_*i*_ denotes the agricultural carbon emission of the ith carbon source

*N*_*i*_ denotes the input of the ith carbon source

*K*_*i*_ is the carbon emission coefficient of the ith carbon source

Table 2 is based on relevant studies by the Oak Ridge National Laboratory, the United Nations Intergovernmental Panel on Climate Change, and the Institute of Agricultural Resources and Ecological Environment of Nanjing Agricultural University. The carbon emissions of the six types of agricultural carbon emission sources in each province from 2012 to 2018 were calculated and the total carbon emissions were calculated as a proxy variable for environmental regulation.

**Table 2:** Carbon emission factors for each carbon source.

Carbon source factor	Carbon emission factor
*Fertilizer*	0.8956 kg C·kg-1
*Pesticides*	4.9341 kg C·kg-1
*Agricultural film*	5.1800 kg C·kg-1
*Diesel*	0.5927 kg C·kg-1
*Tillage*	312.60 kg C·hm-2
*Irrigation*	266.48 kg C·hm-2

#### Mediating variable

Large-scale land management (M). Land scale operation refers to the process by which farmers expand the scale of land operation through various legal means, so as to achieve more effective mechanization and intensification of operation and ultimately improve production efficiency. In view of this, this study refers to Zhou Juan’s approach and selects the land transfer rate to characterise land scale operation, i.e., land scale operation = arable land transfer area/contracted arable land area.

#### Control variables

In order to avoid omission of variables causing regression bias, the following variables are used as control variables in this paper: provincial GDP (GDP), representing the level of economic progress of each province; year-end population (POP), representing population size; rural years of education per capita (EDU), representing the level of education of the rural labor force; gross agricultural output value (GVAP), representing the level of agricultural development; government expenditure (PFE), i.e. the share of fiscal expenditure in (PFE), the share of agricultural expenditure, representing the level of government subsidies; and mean air temperature (MAAT), the average annual temperature of each province, representing the innate development conditions of agriculture in each province.

### Model setting

#### Methods and estimations procedures

This study was conducted to examine the impact of environmental regulation on quality agricultural development in China. This paper draws on relevant studies by Ahmed E. M. scholars to use total factor productivity as an indicator of environmental regulation [50], and uses an integrated approach based on parametric analysis for parametric analysis. This approach combines both growth accounting, which is nonparametric, and econometric and nonparametric estimation. This approach will be applied in two steps: the first step is the econometric estimation, which calculates the parameters of the variables, and the second step puts these parameters into the model and calculates the green total factor productivity indicator.

**Fig 3:**
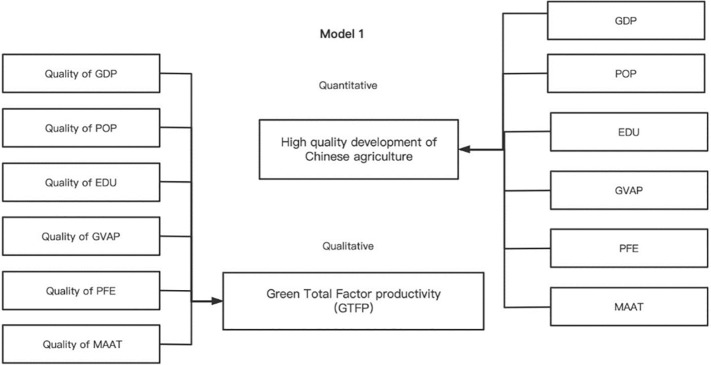
Green productivity framework, extensive growth theory.

The framework (Fig 3) is an introduction to the extensive growth theory of Model (9). China’s high-quality agricultural development is the dependent variable, and provincial GDP (GDP), year-end population (POP), rural years of education per capita (EDU), gross agricultural output (GVAP), government expenditures (PFE), and average air temperature (MAAT) are the variables. In addition, the framework proposes green total factor productivity, expressed as the combined contribution of the quality of environmental regulation (the explanatory variable). Meanwhile, the function of environmental regulation on the quality development of Chinese agriculture can be expressed as follows.

$$Quality_{it}=F(GDP_{it},POP_{it},EDU_{it},GVAP_{it},PFE_{it},MAAT_{it},T_{it})$$ (9)

where Province i = 1, 2,… in years t, high-quality development of Chinese agriculture (Quality) is a function of real fixed physical capital input GDP, POP, EDU, GVAP, PFE, MAAT, and time T that proxies for GTFP as a technological progress of the economies and sustainable development indicator.

#### Extensive growth theory

This subsection presents a broad growth theory based on high-quality agricultural development in China, which is decomposed into provincial GDP (GDP), year-end population (POP), rural years of education per capita (EDU), gross agricultural output (GVAP), government expenditures (PFE), and average air temperature (MAAT). This study attempts to fill this gap by developing this model into a parametric model and providing statistical analysis for it in the first step as follows.

$$\begin{array}{c} \Delta lnQuality_{it}=a+\alpha \cdot \Delta lnGDP_{it}+\beta \cdot \Delta lnPOP_{it}+\lambda \cdot \Delta lnEDU_{it}+\theta \cdot \Delta \text{ln}GVAP_{it}+\\ \mu \cdot \Delta lnPFE_{it}+\sigma \cdot \Delta lnMAAT_{it}+\varepsilon_{it} \end{array}$$ (10)

t = Number of years and i is number of provinces

where

*α* is the output elasticity associated with GDP.

*β* is the output elasticity associated with the number of people at the end of the year.

*λ* is the output elasticity associated with the number of years of education per capita in rural areas.

*θ* is the output elasticity associated with gross agricultural output.

*μ* is the output elasticity associated with government spending.

*σ* is the output elasticity associated with the average temperature.

*a* is the intercept or constant of the model.

*ε* is the residual term.

Ln is the logarithm and is used to convert variables.

Δ is the difference operator that represents the proportional rate of change.

Since the intercept (a) in Eq 2 has no location indicator in the calculation of productivity growth, a second step is proposed. This step calculates the growth rate of the productivity indicator and transforms Eq 2 into an extension of the basic growth accounting framework. The Cobb-Douglas production function is specified in the parameterized form of the above equation as:

$$\begin{array}{c} \Delta lnGTFP_{it}=\Delta lnQuality_{it}-[\alpha \cdot \Delta lnGDP_{it}+\beta \cdot \Delta lnPOP_{it}+\lambda \cdot \Delta lnEDU_{it}\\ +\theta \cdot \Delta \text{ln}GVAP_{it}+\mu \cdot \Delta lnPFE_{it}+\sigma \cdot \Delta lnMAAT_{it}] \end{array}$$ (11)

where the weights are given by the average value shares, as shown below.

Δ*lnQuality*_*it*_ is the growth rate of high-quality agricultural development, *α*∙Δ*lnGDP*_*i*t_ is the contribution of total GDP, *β*∙Δ*lnPOP*_*it*_ is the contribution of the total population, *λ*∙Δ*lnEDU*_*it*_ is the contribution of population education, *θ*∙Δln*GVAP*_*it*_ is the contribution of total agricultural output, *μ*∙Δ*lnPFE*_*it*_ is the contribution of government spending, *σ*∙Δ*lnMAAT*_*it*_ is the Contribution of average temperature, Δ*lnGTFP*_*it*_ is the growth of green total factor productivity. The framework decomposes the growth rate of high-quality agricultural development into the contributions of GDP, year-end population, years of education per rural person, gross agricultural product, government expenditures, and average temperature growth rate, plus a residual of the growth rate commonly referred to as GTFP.

#### Basic model setting

The core problem of this study is to examine the impact of environmental regulation on the Chinese high-quality growth of agriculture. Based on the combination of relevant literature and research, model (12) is constructed as follows. This formula is used to test the impact of environmental regulation on high-quality agricultural development:

$$Quality_{it}=\beta_{0}+\beta_{1}ER_{it}+\beta_{2}CV_{it}+\varepsilon_{it}$$ (12)

where the weights are given by the average value shares as follows:

i reflects the province and t denotes the year
*ER*_*it*_ is the explanatory variable, reflecting the environmental regulation
*Quality*_it_ is the explanatory variable, reflecting the China Agricultural Development Quality Index
*CV*_*it*_ is the control variable
*ε*_*it*_ is the error term

#### Mediating effect model setting

For testing the mechanism of the function of scale operation in the procedure of environmental regulation impacting the quality growth of agriculture, this paper constructs the model shown in Eq (15) for testing, where M represents the mediating variable, i.e., land scale operation.

This formula is used to test the impact of environmental regulation on high-quality agricultural development:

$$Quality_{\text{it}}=\gamma_{0}+\gamma_{1}ER_{\text{it}}+\gamma_{2}CV_{it}+\varepsilon_{it}$$ (13)

This formula is used to test the effect of environmental regulation on mediating variables:

$$M_{it}=\eta_{0}+\eta_{1}ER_{\text{it}}+\eta_{2}CV_{it}+\varepsilon_{it}$$ (14)

This formula is used to test the role of mediating variables:

$$Quality_{\text{it}}=\lambda_{0}+\lambda_{1}ER_{\text{it}}+\lambda_{2}M_{\text{it}}+\lambda_{3}CV_{it}+\varepsilon_{it}$$ (15)

where i reflects the province and t denotes the year

*ER*_*it*_ is the explanatory variable, reflecting the environmental regulation

*Quality*_it_ is the explanatory variable, reflecting the China Agricultural Development Quality Index

*M*_it_ is the mediating variable

*CV*_*it*_ is the control variable

*ε*_*it*_ is the error term

### Data sources and descriptive statistics

#### Data sources

Considering the scientific nature and availability of data, this paper selects provincial panel data of 34 provinces and cities in China from 2012 to 2018 (of which data for Hong Kong, Macau and Taiwan are missing) to study the impact of environmental regulation on high-quality agricultural development. The sample data were mainly obtained from the China Rural Statistical Yearbook, China Statistical Yearbook, National Statistics on Rural Economic Situation, etc. Missing data were supplemented by interpolation and trend extrapolation methods. To address the effects of recording errors and extreme values, the data were subjected to tailoring at the 1% and 99% quantile levels.

#### Descriptive statistics for variables

**Fig 4:**
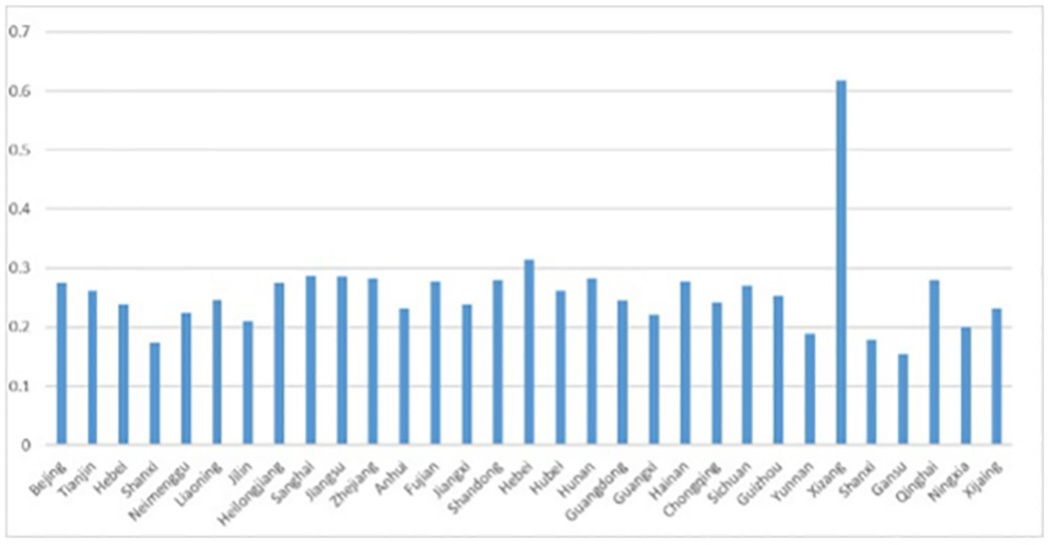
Average index of quality agricultural development by province.

Descriptive statistics for each variable were obtained from data related to the study sample of 34 provinces in China from 2012–2018, analyzed and ranked by statistical software, as shown in Table 3 and Fig 4. The 34 provinces with high agricultural quality development indices during 2012–2018 include Shanghai, Jiangsu, Zhejiang, Henan, Tibet and Qinghai, of which the eastern provinces account for two-thirds, indicating that the provinces with high levels of agricultural quality development during 2012–2018 Most of them are in eastern China. The provinces with a lower agricultural quality development index are Shanxi, Gansu, Ningxia and Jilin, mostly in western China, which indicates that the western region has seen a slower growth in the level of agricultural quality development between 2012 and 2018.

**Table 3:** Descriptive statistics of variables.

*Variable*	Mean	Std.Dev	Min	Max
*Quality*	0.258	0.085	0.130	0.660
*ER*	0.037	0.024	0.001	0.099
*GDP*	2.379	1.912	0.070	9.728
*T-POP*	8.126	0.840	5.730	9.337
*EDU*	7.632	0.830	3.803	9.660
*GVAP*	0.178	0.125	0.0052	0.497
*PFE*	11.559	3.154	4.109	18.960
*MAAT*	13.740	5.453	2.549	25.132

## Empirical analysis

### Unit root test and multicollinearity test

A unit root test and a multicollinearity test were performed for each variable in order to successfully avoid the issue of multicollinearity throughout the model’s design phase. Table 4 shows that the average VIF is 2.65, which is less than 10, indicating that the model has no multicollinearity.

**Table 4:** Multicollinearity test results.

*Variable*	ADFT	VIF
*Quality*	-0.6690***	
*ER*	-0.6389***	2.39
*GDP*	0.0408***	3.49
*T-POP*	-0.0762***	5.20
*EDU*	-0.4407***	1.73
*GVAP*	0.0408***	6.71
*PFE*	-0.4051***	2.64
*MAAT*	-0.4743***	1.54
*Mean VIF*		2.65

Note:

*** means significance at the 1% level.

### Baseline regression analysis

The findings of the model (1) in Table 5 illustrate that the coefficient of the influence of environmental regulation on high-quality growth of agriculture is 1.696 and passes the test at the 1% significance level, pointing out that environmental regulation can suc-cessfully promote the growth of agriculture in China, tentatively proving that hypothesis H1 is valid. The reason may be that with the focus on the implementation of various policies, the efficiency of resource use in agriculture has been improved, production methods have gradually shifted to green and sustainable development has been en-hanced.

**Table 5:** Baseline regression and mediating effect test results.

*Variable*	(1)	(2)	(3)
	*Quality*	*M*	*Quality*
*ER*	1.696***	2.388***	1.386***
	(3.47)	(3.07)	(2.93)
*M*			0.130***
			(3.00)
*GDP*	0.009***	0.021**	0.006
	(2.62)	(1.97)	(1.63)
*T-POP*	-0.080***	0.0189	-0.083***
	(-7.52)	(0.81)	(-7.65)
*EDU*	-0.035***	-0.005	-0.034***
	(-4.76)	(-0.23)	(-4.71)
*GVAP*	0.093	-0.616***	0.173
	(0.77)	(-3.02)	(1.41)
*PFE*	-0.008***	-0.018***	-0.005***
	(-3.85)	(-3.75)	(-2.81)
*MAAT*	0.002***	-0.006***	0.003***
	(2.93)	(-3.38)	(3.80)
*_cons*	1.144***	0.353	1.098***
	(11.58)	(1.27)	(10.50)
*N*	217	217	217
*R* ^*2*^	0.590	0.373	0.631
*adj*. *R*^*2*^	0.564	0.333	0.605

Note:

** means significance at the 5% level;

*** means significance at the 1% level.

As for the control variables, GDP and gross agricultural output value are positively correlated with high-quality agricultural development, indicating that higher GDP and higher gross agricultural output value can produce a good economic environment for high-quality growth of agriculture and can effectively promote its development, which is a crucial driving force for its enhancement in China. The average temperature is posi-tively correlated with the high-quality growth of agriculture, pointing out that the innate natural conditions of a region also have a greater impact on its development. Government spending on agriculture is negatively correlated with high-quality agricultural devel-opment, suggesting that government subsidies for agriculture are not the best and that high investment and subsidies may also make agricultural operators less motivated to foster high-quality growth, which in turn is detrimental to their development. The negative relationship among the rural population and quality agricultural growth may be due to the fact that when there are too many people working in agriculture in a region, agricultural production and agricultural land are overly fragmented, which is not conducive to the use of large agricultural machinery and makes it difficult to promote green technologies in agriculture, thus hindering quality agricultural development.

### Analysis of mediating effects

From Table 5 model (1) it is known that the total effect of environmental regulation on high-quality growth of agriculture is crucial, according to Table 5 model (2) the coefficient of the effect of environmental regulation on land scale operation is 2.388 and is statistically significant at 1% level, highlighting that the environmental regulation helps to promote land scale operation. Finally, by adding both the new environmental regulation and the mechanism variable land scale operation to the equation for estimation, the findings of the model (3) in Table 5 revealed that the coefficient of the effect of environmental regulation on high-quality growth of agriculture was 1.386 and passed the significance test at the 1% level, signifying a significant direct effect; meanwhile, the coefficient of the effect of land scale operation on high-quality growth of agriculture was 0.130 and passed the 1% The coefficient of the effect of large-scale land management on high-quality agricultural development was 0.130, which passed the 1% significance test, showing that the indirect effect was significant. The coefficient of 0.130 for the effect of large-scale land management on high-quality agricultural development passed the 1% significance test, showing an important indirect effect.

### Robustness testing and endogeneity treatment

#### Substitution of core explanatory variables

Considering the variety of measures regarding the intensity of environmental regulation, this study tests the robustness of the conclusions obtained by replacing its measure. The ratio of the change in agricultural chemical oxygen demand (COD) emis-sions to agricultural value added can also reflect environmental regulation, and this study follows the treatment by replacing the measure of the core explanatory variable with the ratio of agricultural-related COD to agricultural value-added in wastewater. The regression findings are presented in the model (1) of Table 6, where the influence of environmental regulation on the quality of agricultural development remains significantly positive after changing the way it is measured. Combined with the above tests, this paper concludes that the empirical results of environmental regulation promoting high-quality growth of agriculture in China are robust.

**Table 6:** Robustness tests.

*Variable*	(1)	(2)
	*Quality*	*Quality*
*ER*	0.743**	1.300**
	(2.05)	(2.27)
*GDP*	0.007	0.011**
	(1.45)	(2.20)
*T-POP*	-0.038***	-0.084***
	(-3.12)	(-6.05)
*EDU*	0.023***	-0.036***
	(3.55)	(-4.17)
*GVAP*	-0.163*	0.206
	(-1.68)	(1.30)
*PFE*	-0.008***	-0.008***
	(-3.94)	(-3.43)
*MAAT*	-0.0004	0.002**
	(-0.35)	(2.46)
*_cons*	0.332***	1.186***
	(4.77)	(9.00)
*N*	217	155
*R* ^*2*^	0.415	0.564
*adj*. *R*^*2*^	0.377	0.530

Note:

* means significance at the 10% level;

** means significance at the 5% level;

*** means significance at the 1% level.

#### Excluding the effects of some factors

In November 2016, the Opinions on Improving the Separation of Ownership, Contracting, and Management Rights of Rural Land proposed the implementation of the "separation of three rights," i.e., the parallel separation of ownership, contracting, and management rights, excluding the effects of some factors. This is a major institutional innovation in rural reform, and the regression results may produce errors. In view of this, this paper excludes the years after the implementation of the Opinions (2017 and 2018) and re-runs the regression on the remaining data. The regression findings are illustrated in the model (2) of Table 6, and the impact coefficients between the two are basically consistent with the baseline regression after the exclusion of the sample regression results. Combined with the above tests, this paper concludes that the empirical results of environmental regulation for high-quality growth of agriculture in China are robust.

#### The problem of endogeneity

Considering that various types of environmental policy making as an external condition do not act on agricultural development in the first instance, the transmission of the impact effect takes some time. In this paper, the one-period lagged environmental regulation variables are used as instrumental variables. The regression results dealing with endogeneity remain largely consistent with the baseline model in the previous section. Table 7 shows that the coefficient values and the direction of action are less different from the benchmark regression values, further validating the robustness of the empirical findings in this study.

**Table 7:** Internal validity test.

*Variable*	(1)
	*Quality*
*L*.*ER*	1.593**
	(2.44)
*Constant*	1.119***
	(11.12)
*Underidentification test*	11.919
*P-Value*	0.0000
*Wald-F*	104.659
*KP Wald-F*	20.441
*Control Variables*	YES
*Observations*	186
*R-squared*	0.594

Note:

** means significance at the 5% level;

*** means significance at the 1% level.

## Further study: Analysis of the threshold effect of per capita income

According to the analysis above, environmental regulation can promote high-quality growth of agriculture, however, there may be certain thresholds to its promotion. In areas with low per capita income, enforcement of laws and regulations is often weak, and there are also problems with information asymmetries. At the same time, the shift towards quality, efficiency, and green sustainability in agriculture imply the use of more advanced production technologies and higher demands on farmers, and the role of environmental regulation in the quality development of agriculture is difficult to fully realize if farmers do not have sufficient funds at hand. Therefore, in order to further investigate whether there is a threshold and non-linear effects, this paper utilizes Hansen’s threshold regression model to construct a threshold effect model of environmental regulation affecting quality agricultural development [51]. If there is only one threshold, the single-threshold model Eq (16) is used, and if there are two thresholds, the model Eq (16) can be extended to a double-threshold model Eq (17). The level of income per rural resident (Income) is set as the threshold variable and the following formula is established.

This formula is used to test for a single threshold effect:

$$Quality_{it}=\delta_{0}+\delta_{1}ER_{it}\times I(Income\leq \tau )+\delta_{2}ER_{it}\times I(Income>\tau )+\delta_{3}CV_{it}+\varepsilon_{it}$$ (16)

This formula is used to test for the double threshold effect:

$$\begin{array}{c} Quality_{\text{it}}=\mu_{0}+\mu_{1}ER_{\text{it}}\times I(Income\leq \tau_{1})+\mu_{2}ER_{\text{it}}\times I(\tau_{1}<Income\leq \tau_{2})\\ +\mu_{3}ER_{\text{it}}\times I(Income>\tau_{2})+\mu_{4}CV_{it}+\varepsilon_{it} \end{array}$$ (17)

where the weights are given by the average value shares as follows:

where i represents the province and t represents the year

*ER*_*it*_ is the explanatory variable

*Quality*_it_ is the explanatory variable

*Income* is the threshold variable

*τ*_1_ is the first threshold value obtained in the estimation of the single threshold model

τ2 is the second threshold value obtained in the dual threshold model

*I*(∙) is an indicator function

*CV*_*it*_ is the control variable

*ε*_*it*_ is the error term

(1) The existence of a threshold effect was tested by the self-help method. The results showed that the per capita income level of rural residents significantly passed the single threshold and failed the double threshold test, so the single threshold was chosen. The regression findings of the thresholds are indicated in Table 8.

**Table 8:** Double threshold test for environmental regulation.

Model	F-Value	P-Value	Critical Value			Threshold	95% Confifidence Interval
			1%	5%	10%		
Single threshold	13.530**	0.043	19.880	12.662	10.140	10.052	[10.046 10.096]
Double threshold	9.370	0.213	35.783	21.163	14.901		

Note:

** means significance at the 5% level.

**Fig 5:**
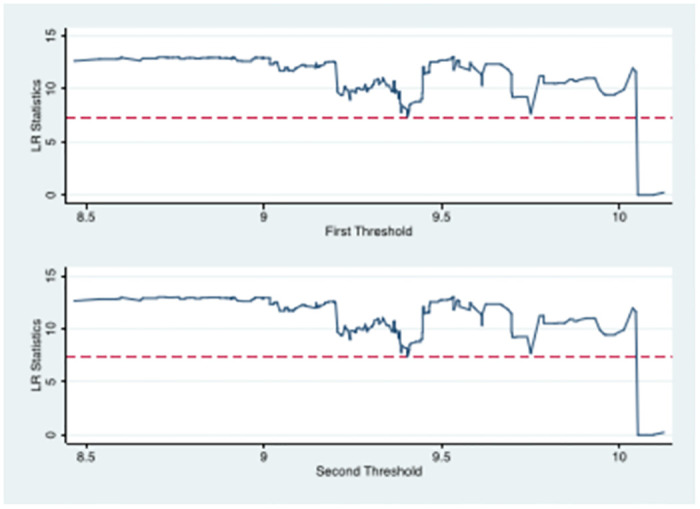
Likelihood ratio function of threshold effect analysis.

(2) Threshold estimates and tests. The threshold estimates and 95% confidence intervals for the income levels of rural residents were determined according to Hansen’s [51] LR test. Table 8 highlights that the single threshold estimate is 10.052 and is within the corresponding 95% confidence interval.

(3) Likelihood ratio function plot for threshold effect analysis. Fig 5 presents the graph of the likelihood ratio function of the threshold. The dashed line indicates the threshold value of 7.35. Since the threshold estimate of the income level of rural residents is smaller than the threshold value, the above estimate is considered to be true and valid.

(4) Estimation findings of the parameters of the threshold model. Based on the above test results, this paper uses a single threshold regression model to empirically test the influence of environmental regulations on high-quality growth of agriculture within different rural residents’ income levels. The regression results in Table 9 depicts that the correlation coefficient between environmental regulation and agricultural quality development is 0.744 at the 1% level in the range of low rural residents’ income levels (Lev≤10.052), indicating that there is a significant positive influence of environmental regulation on agricultural quality development in the case of low rural residents’ income levels. In the range of high rural income levels (Lev>10.052), the correlation coefficient between the two is 1.322 at the 1% level, and the coefficient has increased, and the influence of environmental regulation on high-quality growth of agriculture is gradually increasing. This indicates that the effect of environmental regulation on agricultural quality is to a certain degree limited by the level of disposable income per rural household, and when the income level of rural residents crosses the threshold, the driving power of environmental regulation on the growth of agriculture quality is more stimulated and has a more positive effect.

**Table 9:** Threshold regression results.

*Variable*	*Quanlity*
Income ≤ γ1	0.744***
	(1.76)
Income>γ1	1.322***
	(3.50)
_cons	1.396
	(0.810)
N	217
R^2^	0.1169

Note:

*** means significance at the 1% level.

## Conclusions and implications

### Research conclusion

High-quality agricultural development, which balances efficiency, greenness and sustainability, is a significant direction for the progress of modern agriculture, and identifying the main factors influencing the high-quality growth of agriculture and elucidating the mechanism of action is essential. This study takes environmental regulation as the entry point, uses agricultural data of Chinese provinces from 2012–2018 as the research sample, measures China’s agricultural quality development through the entropy method, and empirically tests of the influence of environmental regulation on (China’s) agricultural quality growth employing an econometric model and a mediating effect model, and uses land scale operation as a mediating variable (to dissect the mechanism of action between the two). Moreover, a threshold model is utilized to empirically examine the non-linear influence of environmental regulation on the high-quality growth of agriculture, which results in the following conclusions.

1. All other things being equal, environmental regulation can significantly aid in the growth of high-quality agriculture in China, and this inference still holds following robustness tests and endogeneity treatments.
2. Regarding mechanism of action, large-scale land management is a critical condition for the high-quality growth of agriculture. Land scale operation not merely receives the influence of environmental regulation, but also affects the quality growth of agriculture. By promoting the scale level of the land, environmental regulations promote its growth.
3. Farmers’ income level is a crucial determinant influencing environmental regulation for fostering the growth of high-quality agriculture in China. There is a single threshold for the influence of environmental regulation on the high-quality growth of agriculture, with a threshold value of 10.052, and when farmers’ income level crosses this threshold value, the function of environmental regulation on the transformation of agricultural level to high-quality is strengthened.

### Management implications

In light of the aforementioned findings, the following recommendations are made.

1. Formulate scientific and reasonable environmental regulation policies and improve the governance level of environmental regulation. China’s current agricultural laws and regulations are relatively imperfect and cannot alleviate the problem of low efficiency and high pollution. From the findings of this study, it can be seen that environmental regulations have positively contributed to the high-quality development of Chinese agriculture. Therefore, the government should be fully aware of the importance of legal policies and actively develop various forms of policy guidelines in practice to reduce surface source pollution. And governments at all levels should gradually improve laws and regulations in line with the actual development of the region, and eventually establish a relatively complete system of laws and regulations to control agricultural pollution, improve agricultural efficiency, and promote the efficient, green and sustainable development of agriculture.
2. Rationalize regulations on land scale management to provide the foundation for high-quality development of Chinese agriculture. China has a predominantly smallholder economy, with frequent land fragmentation and high fertilizer use but low output. From the findings of this study, it can be seen that land scale operation mediates the relationship between environmental regulation and high-quality development of Chinese agriculture. Therefore, it is important to deepen the reform of rural land system and increase the scale of land as much as possible. Actively cultivate new agricultural business entities such as family farms, agricultural enterprises and professional cooperatives to promote the development of traditional single scattered smallholder production to modern agricultural production with scale, intensification and specialization as the main features in order to improve the level of large-scale land management. At the same time, subsidies for large-scale land operation are increased to realize large-scale operation of agricultural land through the optimal combination of input factors, which in turn improves land concentration, fertilizer utilization rate, controls agricultural surface pollution, and creates good conditions for high-quality agricultural development.
3. Improving the income status of Chinese farmers and providing the economic basis for high-quality development of Chinese agriculture. The finding of income as an important threshold in this study shows that for farmers, income is an important factor that affects their ability to accept environmental regulations. Therefore, on the one hand, it is necessary to increase subsidies by raising farmers’ income. Mobilizing the initiative, initiative and creative output of farmers in all areas of production and life, forming a system of income growth that meets the intrinsic needs of farmers and enhances their ability to generate income, giving them the capital and ability to change production methods, reduce pollution emissions, improve production efficiency and achieve high-quality agricultural development. Finally, based on agriculture and rural areas, we will establish a sound mechanism to increase income within agriculture and lay a solid industrial foundation to ensure that farmers can obtain long-term stable income growth. It is also necessary to create conditions for farmers to carry out specialized, intensive, large-scale and socialized production and operation and agricultural services, and to increase the property income and wage income of some farmers, so that they are willing and able to contribute their share to the high-quality growth of Chinese agriculture.

### Considerations and future research

This paper also has certain drawbacks. First, the indications created are insufficiently detailed. Given the availability of data, we established certain measures to assess the quality of agricultural development, although these indicators are insufficiently precise. When data becomes accessible, we should monitor agricultural quality development from additional viewpoints and in more depth in the future. Second, the influence of environmental legislation on agriculture has not been well researched. Although environmental control has a substantial impact on agricultural quality improvement, it may also have some negative consequences, and excessive environmental regulation may demotivate farmers to produce. As a result, the negative impact of environmental regulation on agriculture may be investigated in the future, both to mitigate the projected unfavorable scenario and to enhance the fit between environmental regulation and agricultural productivity. Third, the impact mechanisms are insufficiently developed. This paper examined the impact of environmental regulation on high-quality agricultural development primarily from the perspective of land scaling; however, the impact of environmental regulation is extensive, and the factors influencing high-quality agricultural development are constantly expanding.
